# Cross-cultural assessment of knowledge and attitudes toward Folic acid: Instrument development and validation in Thailand and Yemen

**DOI:** 10.1371/journal.pone.0352966

**Published:** 2026-07-15

**Authors:** Rana Ali Al-Qaari, Saranthorn Khuankaew, Mahittha Suttiserm, Siraphat Pornsuksawang, Pawat Boonnop, Somchai Manopatanakul, Siribang-on Piboonniyom Khovidhunkit, Sasipa Thiradilok, Chanita Tantipoj

**Affiliations:** 1 Department of Advanced General Dentistry, Faculty of Dentistry, Mahidol University, Bangkok, Thailand; 2 Faculty of Dentistry, Mahidol University, Bangkok, Thailand; Southern Illinois University Carbondale, UNITED STATES MINOR OUTLYING ISLANDS

## Abstract

Folic acid is essential for the prevention of neural tube defects and other congenital anomalies, yet awareness and uptake vary across cultural and resource settings. Reliable tools to assess women’s knowledge and attitudes toward Folic acid are limited, particularly in cross-cultural contexts. This study aimed to develop and validate a culturally adaptable questionnaire in two distinct populations: Thailand and Yemen. In this cross-sectional instrument development and validation study, the questionnaire was developed through literature review and expert consultation, and comprised demographics, eight knowledge items, and ten attitude items. Following content validation, pilot testing, and test–retest reliability assessment, exploratory and confirmatory factor analyses were performed. Cronbach’s alpha was calculated for internal consistency. Data were collected from 538 women of reproductive age (Thai: 104; Yemeni: 434) via an online self-administered survey. The attitude scale demonstrated strong psychometric properties, with high internal consistency (α = 0.88 in both groups), coherent factor structures, and excellent model fit in the Yemeni sample (RMSEA = 0.054, CFI = 0.973, TLI = 0.965). Knowledge scores varied across items, with higher awareness in dietary source and timing-related items, and lower scores in statistical and biological knowledge. The Yemeni version showed lower internal consistency in the knowledge section (α = 0.59), reflecting content heterogeneity. Despite contextual differences, the instrument demonstrated psychometric coherence in both groups, though model fit was more robust in the Yemeni sample. In conclusion, this study developed and validated a brief questionnaire to assess knowledge and attitudes toward Folic acid in culturally diverse settings. The tool showed good psychometric performance and is suitable for use in research and public health interventions, particularly in low-resource or cross-cultural environments.

## Introduction

Folic acid (vitamin B9) plays a key role in preventing neural tube defects and other congenital anomalies. In many countries, public health policies have promoted Folic acid supplementation or food fortification to address deficiencies, particularly among women of childbearing age [1]. However, awareness, accessibility, and behavioral uptake of Folic acid remain uneven—especially in resource-limited or culturally diverse contexts [2,3].

Understanding women’s knowledge and attitudes toward Folic acid is essential for designing effective interventions and informing public health strategies. Yet, despite its importance, most existing studies have employed self-developed or adapted questionnaires without rigorous psychometric validation [4,5]. Many of these tools either emphasize biomedical knowledge or rely on simplified behavioral indicators, with limited attention to cultural adaptation or measurement reliability [6,7].

While some tools have been developed to assess knowledge or attitudes toward Folic acid in specific populations, many lack comprehensive validation across diverse settings. Prior studies have primarily targeted Western or high-income country populations, with limited application in multilingual or low-resource contexts [4,6]. Moreover, few instruments have undergone both exploratory and confirmatory factor analyses, test-retest reliability testing, or careful linguistic adaptation [5,7]. This leaves a gap in the availability of validated tools for cross-cultural use, particularly in fragile or underserved populations.

This study aimed to develop and validate a culturally adaptable questionnaire to assess women’s knowledge and attitudes toward Folic acid. We tested the instrument in two culturally and structurally distinct countries: Thailand and Yemen. These settings differ widely in language, culture, healthcare infrastructure, and digital access, offering a unique opportunity to examine the robustness and adaptability of the tool. Yemen, in particular, represents a hard-to-reach population where public health research tools are scarce [8]. This diversity enabled the instrument to be evaluated not only for psychometric quality but also for its cross-contextual flexibility. Special attention was given to the attitude domain, where belief systems may be culturally influenced and harder to measure reliably, in contrast to factual knowledge that may vary by exposure or education.

## Materials and methods

### Study design and participants

This cross-sectional instrument development and validation study consisted of three phases: (1) pilot testing, (2) validation in the Thai population, and (3) cross-cultural validation in the Yemeni population. Participants were recruited via online platforms in both settings using convenience sampling. The Thai dataset had been collected previously and was retrieved in October 2025 for secondary analysis in the present study. Data for the Yemeni sample were collected between December 2023 and April 2024.

Ethical approval for both datasets was obtained from the Mahidol University Institutional Review Board (COE.No.MU-DT/PY-IRB 2022/039.1808), which granted an exemption from full review. The study was conducted in accordance with the principles of the Declaration of Helsinki. Participants were informed about the study objectives, and consent was implied by their voluntary completion of the questionnaire. Additional information regarding ethical, cultural, and scientific considerations specific to inclusivity in global research is provided in the Supporting Information (S1 Checklist).

In this study, the Thai sample comprised 104 women aged 18–40 years, recruited through Facebook groups related to motherhood and childcare. The Yemeni sample included 434 women residing in Sana’a. Yemeni participants were recruited through women’s community WhatsApp groups, where the Yemeni co-author (RAA), a native speaker and community member, had established appropriate access to these communities. The involvement of a Yemeni researcher facilitated culturally appropriate communication, recruitment, and interpretation of the survey. Permission from group administrators enabled feasible and culturally appropriate distribution of the survey link. Yemen was selected because women’s access to antenatal care, nutrition information, and Folic-acid–related health resources is limited in this conflict-affected setting. Including this population also allowed the instrument to be evaluated in a linguistically and culturally distinct context, supporting cross-cultural validation of the questionnaire.

Sample size justification followed established recommendations for factor-analytic studies, which suggest at least 5–10 participants per item [9]. With 10 attitude items, the Thai dataset (n = 104) met this requirement, while the Yemeni dataset (n = 434) provided a sufficiently large sample for robust validation.

### Questionnaire development

The questionnaire was developed through a literature-informed process, drawing from sources such as the World Health Organization (WHO), PubMed-indexed studies, and national health databases. It was designed to explore three domains: demographics, knowledge, and attitudes related to Folic acid. The demographic section included five items: age, marital status, education, occupation, and prior awareness of Folic acid.

To assess knowledge, eight dichotomous items (scored as correct = 1, incorrect = 0) were developed, focusing on dietary sources, timing of consumption, recommended dosage, and the nutrient’s role in preventing birth defects. Some items also addressed broader topics, including awareness of population-level statistics and countries implementing mandatory food fortification policies. One knowledge item on the recommended daily intake of 5 mg of Folic acid was based on national guidelines for women at high risk of neural tube defects, reflecting context-specific practices. In addition, certain knowledge items were adapted to reflect country-specific public health contexts, such as national statistics and locally relevant information, while maintaining conceptual equivalence across settings. Knowledge items were therefore analyzed descriptively and interpreted within the context of each setting.

Attitudes toward Folic acid were measured using ten statements rated on a 5-point Likert scale. These items captured willingness to take Folic acid, support for public distribution programs, and views on government policies related to accessibility. An example item was: “I agree that women of childbearing age should consume Folic acid regularly.”

### Translation and adaptation

The questionnaire was initially composed in Thai, translated into Arabic by professional translators, and reviewed by bilingual health professionals to ensure conceptual alignment and linguistic clarity [10].

### Content validity and pilot testing

Three subject matter experts—a dental public health specialist, a pedodontist, and an orthodontist—evaluated content validity. Items were revised until all reached an item-objective congruence (IOC) score greater than 0.5 [11].

A pilot test with 30 Thai women assessed clarity, feasibility, and preliminary reliability. Cohen’s Kappa was used for dichotomous knowledge items and weighted Kappa for Likert-scale attitude items. Agreement scores ranged from moderate to substantial [12].

### Construct validity

Exploratory factor analysis (EFA) was used to examine the latent structure of the attitude items. Sampling adequacy was confirmed using the Kaiser-Meyer-Olkin (KMO) test and Bartlett’s test of sphericity. Factors were extracted using eigenvalues >1 and scree plot inspection. Items with factor loadings ≥ 0.40 were retained [13,14].

Confirmatory factor analysis (CFA) was then performed to confirm the structure identified in EFA using the same dataset within each country sample. Model fit was evaluated using standard thresholds: RMSEA ≤0.08, CFI ≥ 0.90, and χ²/df < 3. The Thai model showed poor fit, while the Yemeni model demonstrated good fit after removal of two weak-loading items [15].

### Internal consistency

Cronbach’s alpha was used to assess internal consistency. The attitude section showed strong reliability in both populations (α > 0.87). The knowledge section showed acceptable reliability in the Thai sample (α = 0.73), but lower reliability in the Yemeni sample (α = 0.59) [16].

### Statistical analysis

Data were analyzed using Stata version 18. Descriptive statistics were computed for demographic characteristics and questionnaire responses. Analyses were conducted using available data for each item; therefore, the sample size may vary slightly across items due to missing responses. Missing data were minimal (<1%) and were handled using complete-case analysis without imputation. Psychometric properties of the questionnaire were evaluated using established validation procedures. Test–retest reliability was assessed using Cohen’s Kappa for dichotomous knowledge items and weighted Kappa for Likert-scale attitude items. Exploratory factor analysis (EFA) was performed to examine the latent structure of the attitude items, and confirmatory factor analysis (CFA) was subsequently conducted to evaluate the model fit of the identified factor structure. Details of the factor extraction criteria are described in the Construct Validity section. Internal consistency was assessed using Cronbach’s alpha. Thai and Yemeni datasets were analyzed separately to preserve cultural relevance.

### Inclusivity in global research

Additional information regarding ethical, cultural, and scientific considerations related to inclusivity in global research is provided in the Supporting Information (S1 Checklist).

## Results

### Demographic characteristics

Table 1 presents the demographic characteristics of participants from Thailand (n = 104) and Yemen (n = 434). Most Yemeni respondents were aged 18–34 years (94.4%), while the Thai sample showed a broader age distribution. A similar proportion of single participants was observed in both groups, slightly higher among Thai respondents (74.0% vs. 67.5%).

**Table 1:** Demographic characteristics of Yemeni and Thai participants.

Variable	Thai (104)	Yemen (434)
	n (%)	n (%)
Age		
18-23	25 (24.0)	192 (44.2)
24-34	54 (52.0)	197 (45.4)
35-40	25 (24.0)	45 (10.4)
Marriage Status		
Single	77 (74.0)	293 (67.5)
Married/ Divorce	27 (26.0)	141 (32.5)
Education level		
High school and less	7 (6.7)	70 (16.1)
Technical education and diploma	1 (1.0)	65 (15.0)
Undergraduate degree	83 (79.8)	230 (53.0)
Postgraduate degree	13 (12.5)	37 (8.5)
Prefer not to answer	0 (0.0)	32 (7.4)
Occupation		
Unemployed	2 (1.9)	74 (17.1)
Student	25 (24.0)	254 (58.5)
Government officer	26 (25.0)	26 (6.0)
Non-government officer	50 (48.1)	70 (16.1)
Prefer not to answer	1 (1.0)	10 (2.3)
Have you ever heard about Folic acid?		
Yes	70 (67.3)	388 (89.4)
No	34 (32.7)	46 (10.6)

Higher educational attainment was more common in the Thai group (92.3% vs. 61.5%). Most Yemeni participants were students (58.5%). Prior awareness of Folic acid was reported by 89.4% of Yemeni and 67.3% of Thai participants. These differences may reflect variations in education access, health messaging exposure, or recruitment contexts.

### Test-retest reliability

Test–retest reliability was assessed among 30 Thai participants using Cohen’s and weighted Kappa statistics. Knowledge items yielded Kappa values ranging from 0.65 to 0.82 (mean = 0.74), indicating moderate to substantial agreement. Attitude items demonstrated stronger reliability, with values between 0.75 and 0.86 (mean = 0.80), indicating substantial to almost perfect agreement. No item fell below the accepted threshold of 0.60 [17]. Item-level statistics are presented in S1 Table.

### Exploratory factor analysis

**Fig 1:**
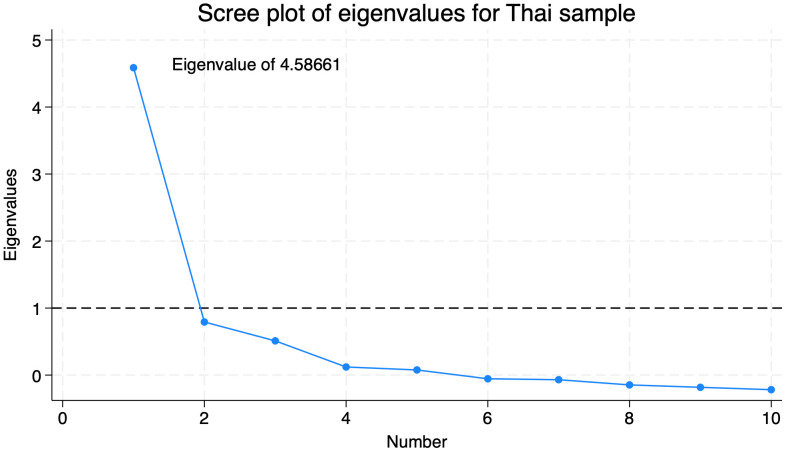
Scree plot for Thai population (n = 104).

**Fig 2:**
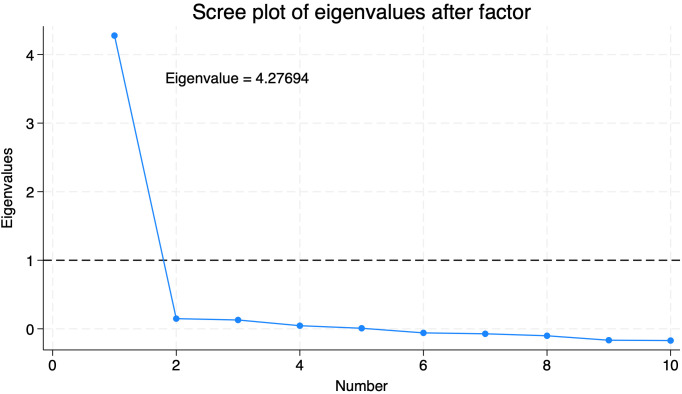
Scree plot for Yemeni population (full model, n = 434).

Exploratory factor analysis (EFA) supported a single-factor structure for the attitude items in both Thai and Yemeni populations. In the Thai sample, the scree plot revealed a steep drop after the first component (eigenvalue = 4.59), indicating a unidimensional construct (Fig 1). In the Yemeni dataset, the initial 10-item model produced an eigenvalue of 4.28 for the first factor (Fig 2). After removing two items with low factor loadings (<0.40), the reduced 8-item model preserved the one-factor structure (S1 Fig).

The final Yemeni model retained 8 items, each showing moderate to strong loading (0.60–0.82). Uniqueness values ranged from 0.39 to 0.64, indicating that a substantial proportion of variance in each item was explained by the latent construct (Table 2).

**Table 2:** Factor loadings and uniqueness for the reduced 8-item model (Yemeni sample).

Item	Factor Loading (Yemen)	Factor Loading (Yemen RM)	Uniqueness
Att1. You agree that women of childbearing age should consume Folic acid	0.6772	0.6675	0.5545
Att2. You will choose foods and beverages that contain Folic acid, although they are more expensive than ones that do not contain Folic acid	0.4226	–	–
Att3. If Folic acid consumption is recommended, you will not hesitate to follow the advice	0.6209	0.6093	0.6288
Att4. You agree that there should be law enforcing Folic acid fortified to main staple such as rice	0.5163	–	–
Att5. You agree that Folic acid consumption from pre-pregnancy to the first 3 months of pregnancy benefits more than being harmful	0.6577	0.666	0.5565
Att6. Consuming Folic acid during pregnancy can prevent baby birth defects	0.6064	0.6035	0.6358
Att7. If you are planning pregnancy, you will purchase and take Folic acid	0.7811	0.7839	0.3855
Att8. You agree if free Folic acid is given to women of childbearing age	0.7513	0.7618	0.4197
Att9. Folic acid is readily available at most drugstores	0.7103	0.7151	0.4886
Att10. To improve the Folic acid accessibility to the public, you agree with the government program to support the distribution of Folic acid	0.713	0.7064	0.5009

Note: Factor loadings ≥ 0.40 were considered acceptable. Uniqueness represents the proportion of variance in each item not explained by the factor. Items 2 and 4 were excluded in the reduced model due to high uniqueness (>0.70), despite meeting the minimum loading threshold.

### Confirmatory factor analysis

Confirmatory factor analysis (CFA) was performed to assess the structural validity of the attitude scale in both the Thai and Yemeni samples. The Thai model yielded poor fit indices (RMSEA = 0.162, CFI = 0.810, TLI = 0.756, SRMR = 0.091). In contrast, the full Yemeni model demonstrated good fit (RMSEA = 0.054, CFI = 0.973, TLI = 0.965, SRMR = 0.032). After removing two weak-loading items, the reduced 8-item model retained acceptable fit across all indices (RMSEA = 0.065, CFI = 0.974, TLI = 0.964, SRMR = 0.031), indicating good structural robustness [18] (Table 3).

**Table 3:** Confirmatory factor analysis fit indices for attitude models in Thai and Yemeni samples.

Fit Index	Thai	Yemen (Full model)	Yemen (Reduce model)	Threshold
χ² (Chi-square)	129.062	78.936	56.592	*P* > 0.05
RMSEA	0.162	0.054	0.065	≤ 0.08
CFI	0.810	0.973	0.974	≥ 0.90
TLI	0.756	0.965	0.964	≥ 0.90
SRMR	0.091	0.032	0.031	≤ 0.08

Fit indices were interpreted using established cutoffs: RMSEA ≤ 0.08, CFI and TLI ≥ 0.90, SRMR ≤ 0.08. The reduced Yemeni model demonstrated the best overall fit across all indicators.

### Internal consistency

Internal consistency was assessed using Cronbach’s alpha. For the knowledge section, the Thai version showed acceptable reliability (α = 0.73), while the Yemeni version showed a lower alpha (α = 0.59) [16]. In contrast, the attitude section demonstrated strong reliability across both populations (α = 0.88). The reduced Yemeni model also retained the same reliability (α = 0.88).

### Knowledge scores

Item-level analysis of knowledge scores revealed variation across specific topics (Table 4). The highest mean scores were observed for natural dietary sources (K3: Thai = 0.83, Yemen = 0.71) and recommended timing of supplementation (K5: Thai = 0.66, Yemen = 0.78). The lowest scores were found for population-level statistics (K1) and Folic acid excretion (K7), particularly among Yemeni participants. These findings suggest that while foundational knowledge of Folic acid was moderately high, gaps remained in technical or policy-related areas.

**Table 4:** Knowledge scores by item among Thai and Yemeni participants.

Question	Thai		Yemeni	
	Mean	SD	Mean	SD
K1. Approximately [country-specific number] children are born with disabilities each year. *(True)*	0.29	0.46	0.44	0.50
K2. Cleft lip and cleft palate are not considered congenital defects. *(False)*	0.72	0.45	0.68	0.47
K3. Folic acid is found in both food sources and dietary supplements. *(True)*	0.83	0.38	0.71	0.46
K4. Currently, many countries add Folic acid to staple foods such as rice. *(True)*	0.36	0.48	0.45	0.50
K5. Folic acid should be consumed from the pre-pregnancy period through the first three months of pregnancy. *(True)*	0.66	0.47	0.76	0.43
K6. To reduce the risk of birth defects, pregnant women should consume 5 mg of Folic acid daily. *(True)*	0.49	0.50	0.51	0.50
K7. Folic acid cannot be excreted from the body. *(False)*	0.53	0.50	0.27	0.45
K8. Only women of childbearing age can take Folic acid. *(False)*	0.63	0.49	0.63	0.48

Note: The expected correct answer for each item is indicated in parentheses.

### Attitude scores

Both groups demonstrated generally positive attitudes across all 10 items (Table 5). The highest agreement was observed for policy- and accessibility-related items, such as supporting government programs for Folic acid distribution (Att10: Thai = 4.50, Yemen = 4.48) and endorsing the provision of free Folic acid (Att8: Thai = 4.49, Yemen = 4.34). Thai participants reported lower scores for willingness to purchase Folic-rich foods despite higher prices (Att2: 3.48) and for regulatory support such as enforcing food fortification (Att4: 3.35) compared to the Yemeni group. These trends may reflect cultural or contextual differences in health literacy and accessibility.

**Table 5:** Attitude scores by item among Thai and Yemeni participants.

Question	Thai		Yemen	
	Mean	SD	Mean	SD
Att1. You agree that women of childbearing age should consume Folic acid	4.42	0.75	4.37	0.80
Att2. You will choose foods and beverages that contain Folic acid, although they are more expensive than ones that do not contain Folic acid	3.48	1.09	4.08	0.84
Att3. If Folic acid consumption is recommended, you will not hesitate to follow the advice	3.86	0.97	4.33	0.78
Att4. You agree that there should be law enforcing Folic acid fortified to main staple such as rice	3.35	1.07	3.93	0.92
Att5. You agree that Folic acid consumption from pre-pregnancy to the first 3 months of pregnancy benefits more than being harmful	4.39	0.85	4.21	0.82
Att6. Consuming Folic acid during pregnancy can prevent baby birth defects	4.38	0.84	4.23	0.84
Att7. If you are planning pregnancy, you will purchase and take Folic acid	4.24	0.97	4.31	0.83
Att8. You agree if free Folic acid is given to women of childbearing age	4.49	0.75	4.34	0.86
Att9. Folic acid is readily available at most drugstores.	4.27	0.87	4.44	0.71
Att10. To improve the Folic acid accessibility to the public, you agree with the government program to support the distribution of Folic acid	4.5	0.70	4.48	0.67

## Discussion

This study developed and tested a questionnaire to assess women’s knowledge and attitudes toward Folic acid in Thai and Yemeni populations. The instrument showed strong psychometric properties, including acceptable content validity, high internal consistency for the attitude section, and factor structures supported by both EFA and CFA. Despite cultural and contextual differences, the final attitude model demonstrated good fit in both groups. These findings align with best-practice guidelines for cross-cultural instrument development, which emphasize adaptation, pretesting, and validation through EFA and CFA [19,20].

The questionnaire’s strength lies in its rigorous design. Items were drawn from the literature and refined by expert review, ensuring conceptual clarity and contextual relevance [21]. Pilot testing confirmed feasibility and comprehension. Reliability was supported by Kappa statistics and Cronbach’s alpha. The attitude scale achieved strong performance in both groups, with good model fit in Yemen and a coherent structure in Thailand. The weaker CFA fit in Thailand was likely due to the smaller sample size, highlighting the need for further testing in larger Thai cohorts [22].

Validation in two contrasting contexts underscores the instrument’s transferability. The Thai and Yemeni samples were drawn from markedly different sociocultural contexts. Thailand represents a middle-income country with relatively broad digital health access, whereas Yemen has faced prolonged conflict and resource limitations [23]. Despite these contrasts, the tool performed consistently, suggesting that careful linguistic and cultural adaptation may help preserve validity across different contexts. The substantial participation of Yemeni women also suggests that the tool may be feasible in similar underrepresented populations using online survey approaches.

The distribution of factor loadings across the retained attitude items also suggests a relatively stable factor structure. As illustrated in S2 Fig, all eight retained items showed factor loadings above 0.40, with values ranging from 0.60 to 0.78. The relatively similar magnitude of these loadings indicates that multiple items contributed to the factor structure.

Several limitations must be considered. First, the small Thai sample size may have affected CFA fit. Second, participants were informed that the survey focused on Folic acid, which may have introduced response bias. Third, the knowledge section showed lower internal consistency in Yemen, reflecting the diverse and factual nature of items; such items are not expected to form a single construct [16]. Fourth, EFA and CFA were conducted using the same dataset within each country sample, which may increase the risk of model overfitting; future studies with independent samples are recommended to further validate the factor structure. Finally, online self-administered surveys may have excluded women with limited digital literacy or internet access, particularly in Yemen [8].

Overall, this questionnaire provides a reliable and adaptable tool for assessing knowledge and attitudes toward Folic acid. Its consistent psychometric performance suggests potential application in other low- and middle-income settings, where validated tools for public health assessment are often lacking.

## Conclusion

This study developed and validated a questionnaire to assess women’s knowledge and attitudes toward Folic acid in Thailand and Yemen. The instrument showed good psychometric properties, including acceptable reliability and validity across both settings. Its consistent performance suggests that the tool is suitable for use in diverse cultural contexts and may support future public health research and interventions in low- and middle-income countries.

## Supporting information

S1 Table Test–retest reliability for knowledge and attitude items using Cohen’s Kappa among 30 Thai participants. (DOCX)

S2 Table Factor loadings and uniqueness for the reduced 8-item attitude model (Yemeni sample). (DOCX)

S3 Table Confirmatory factor analysis fit indices for attitude models in Thai and Yemeni samples. (DOCX)

S1 Fig Scree plot for the Yemeni sample, reduced 8-item model (n = 434). (TIFF)

S2 Fig Radar chart showing the standardized factor loadings of the eight attitude items derived from exploratory factor analysis in the Yemeni sample. The red line represents the loading threshold of 0.40. All items exceeded this threshold, indicating adequate contribution to the underlying construct.
(PDF)

S1 Questionnaire Thai version (CC BY 4.0). (DOCX)

S2 Questionnaire Arabic version (CC BY 4.0). (DOCX)

S3 Questionnaire English version (CC BY 4.0). (DOCX)

S1 Dataset Minimal anonymized dataset used for statistical analysis. (XLSX)
