# Supplementary material for: Cross-cultural assessment of knowledge and attitudes toward Folic acid: Instrument development and validation in Thailand and Yemen
Source: PLoS One. 2026 Jul 15;21(7):e0352966. doi: 10.1371/journal.pone.0352966 (PMC13372155; doi:10.1371/journal.pone.0352966)
Supplement: S1 Table — (DOCX) [file pone.0352966.s001.docx]

| **Item** | **Kappa Value** | **Interpretation** |
| --- | --- | --- |
| Knowledge Items |  |  |
| K1. Approximately 30,000 children are born with disabilities each year. | 0.79 | Substantial |
| K2. Cleft lip and cleft palate are not considered congenital defects. (False) | 0.82 | Almost perfect |
| K3. Folic acid is found in both food sources and dietary supplements. | 0.76 | Substantial |
| K4. Currently, many countries add Folic acid to staple foods such as rice. | 0.74 | Substantial |
| K5. Folic acid should be consumed from the pre-pregnancy period through the first three months of pregnancy. | 0.68 | Substantial |
| K6. To reduce the risk of birth defects, pregnant women should consume 5 mg of Folic acid daily. | 0.71 | Substantial |
| K7. Folic acid cannot be excreted from the body. *(False)* | 0.65 | Moderate |
| K8. Only women of childbearing age can take Folic acid. *(False)* | 0.77 | Substantial |
| Attitude Items |  |  |
| Att1. You agree that women of childbearing age should consume Folic acid | 0.83 | Almost perfect |
| Att2. You will choose foods and beverages that contain Folic acid, although they are more expensive than ones that do not contain Folic acid | 0.85 | Almost perfect |
| Att3. If Folic acid consumption is recommended, you will not hesitate to follow the advice | 0.81 | Almost perfect |
| Att4. You agree that there should be law enforcing Folic acid fortified to main staple such as rice | 0.78 | Substantial |
| Att5. You agree that Folic acid consumption from pre-pregnancy to the first 3 months of pregnancy benefits more than being harmful | 0.75 | Substantial |
| Att6. Consuming Folic acid during pregnancy can prevent baby birth defects | 0.84 | Almost perfect |
| Att7. If you are planning pregnancy, you will purchase and take Folic acid | 0.69 | Substantial |
| Att8. You agree if free Folic acid is given to women of childbearing age | 0.86 | Almost perfect |
| Att9. Folic acid is readily available at most drugstores | 0.8 | Almost perfect |
| Att10. To improve the Folic acid accessibility to the public, you agree with the government program to support the distribution of Folic acid | 0.79 | Substantial |

**S1 Table. Test–retest reliability for knowledge and attitude items using Cohen’s Kappa among 30 Thai participants.**

Note: Interpretation of Kappa values follows Landis and Koch (1977): <0.00 = Poor; 0.00–0.20 = Slight; 0.21–0.40 = Fair; 0.41–0.60 = Moderate; 0.61–0.80 = Substantial; 0.81–1.00 = Almost perfect agreement.
