# Supplementary material for: Cross-cultural assessment of knowledge and attitudes toward Folic acid: Instrument development and validation in Thailand and Yemen
Source: PLoS One. 2026 Jul 15;21(7):e0352966. doi: 10.1371/journal.pone.0352966 (PMC13372155; doi:10.1371/journal.pone.0352966)
Supplement: S2 Table — (DOCX) [file pone.0352966.s002.docx]

| **Knowledge Item** | **Thai  % Correct** | **Yemen % Correct** | **Thai Mean (SD)** | **Yemen Mean (SD)** |
| --- | --- | --- | --- | --- |
| K 1. Approximately [country-specific number] children are born with disabilities each year. | 28.9 | 44.5 | 0.29 (0.46) | 0.44 (0.50) |
| K 2. Cleft lip and cleft palate are not considered congenital defects. (False) | 72.1 | 67.7 | 0.72 (0.45) | 0.68 (0.47) |
| K 3. Folic acid is found in both food sources and dietary supplements. | 82.7 | 70.7 | 0.83 (0.38) | 0.71 (0.46) |
| K 4. Currently, many countries add Folic acid to staple foods such as rice. | 35.6 | 45.1 | 0.36 (0.48) | 0.45 (0.50) |
| K 5. Folic acid should be consumed from the pre-pregnancy period through the first three months of pregnancy. | 66.4 | 76.3 | 0.66 (0.47) | 0.76 (0.43) |
| K 6. To reduce the risk of birth defects, pregnant women should consume 5 mg of Folic acid daily. | 49 | 50.9 | 0.49 (0.50) | 0.51 (0.50) |
| K 7. Folic acid cannot be excreted from the body. *(False)* | 52.9 | 27.4 | 0.53 (0.50) | 0.27 (0.45) |
| K 8. Only women of childbearing age can take Folic acid. *(False)* | 62.5 | 63.1 | 0.63 (0.49) | 0.63 (0.48) |

**S2 Table. Item-level mean scores and percentage of correct responses for knowledge items among Thai and Yemeni participants.**

Note: The expected correct answer for each item is indicated in parentheses.
