# Supplementary material for: Cross-cultural assessment of knowledge and attitudes toward Folic acid: Instrument development and validation in Thailand and Yemen
Source: PLoS One. 2026 Jul 15;21(7):e0352966. doi: 10.1371/journal.pone.0352966 (PMC13372155; doi:10.1371/journal.pone.0352966)
Supplement: S3 Table — (DOCX) [file pone.0352966.s003.docx]

| **Attitude Item** | **Factor Loading (Thai)** | **Uniqueness** |
| --- | --- | --- |
| Att1. You agree that women of childbearing age should consume Folic acid | 0.6671 | 0.5549 |
| Att2. You will choose foods and beverages that contain Folic acid, although they are more expensive than ones that do not contain Folic acid | 0.5727 | 0.672 |
| Att3. If Folic acid consumption is recommended, you will not hesitate to follow the advice | 0.621 | 0.6144 |
| Att4. You agree that there should be law enforcing Folic acid fortified to main staple such as rice | 0.492 | 0.758 |
| Att5. You agree that folic acid consumption from pre-pregnancy to the first 3 months of pregnancy benefits more than being harmful | 0.641 | 0.5891 |
| Att6. Consuming folic acid during pregnancy can prevent baby birth defects | 0.7479 | 0.4406 |
| Att7. If you are planning pregnancy, you will purchase and take Folic acid | 0.7167 | 0.4863 |
| Att8. You agree if free Folic acid is given to women of childbearing age | 0.6933 | 0.5194 |
| Att9. Folic acid is readily available at most drugstores. | 0.8063 | 0.3499 |
| Att10. To improve the folic acid accessibility to the public, you agree with the government program to support the distribution of Folic acid | 0.7558 | 0.4288 |

**S3 Table. Exploratory factor analysis results for attitude items among Thai participants (n = 104).**

Note: Factor loadings ≥0.40 were considered acceptable. Uniqueness represents the proportion of variance not explained by the factor.
