# Supplementary figures and images for: Cross-cultural assessment of knowledge and attitudes toward Folic acid: Instrument development and validation in Thailand and Yemen
Source: PLoS One. 2026 Jul 15;21(7):e0352966. doi: 10.1371/journal.pone.0352966 (PMC13372155; doi:10.1371/journal.pone.0352966)

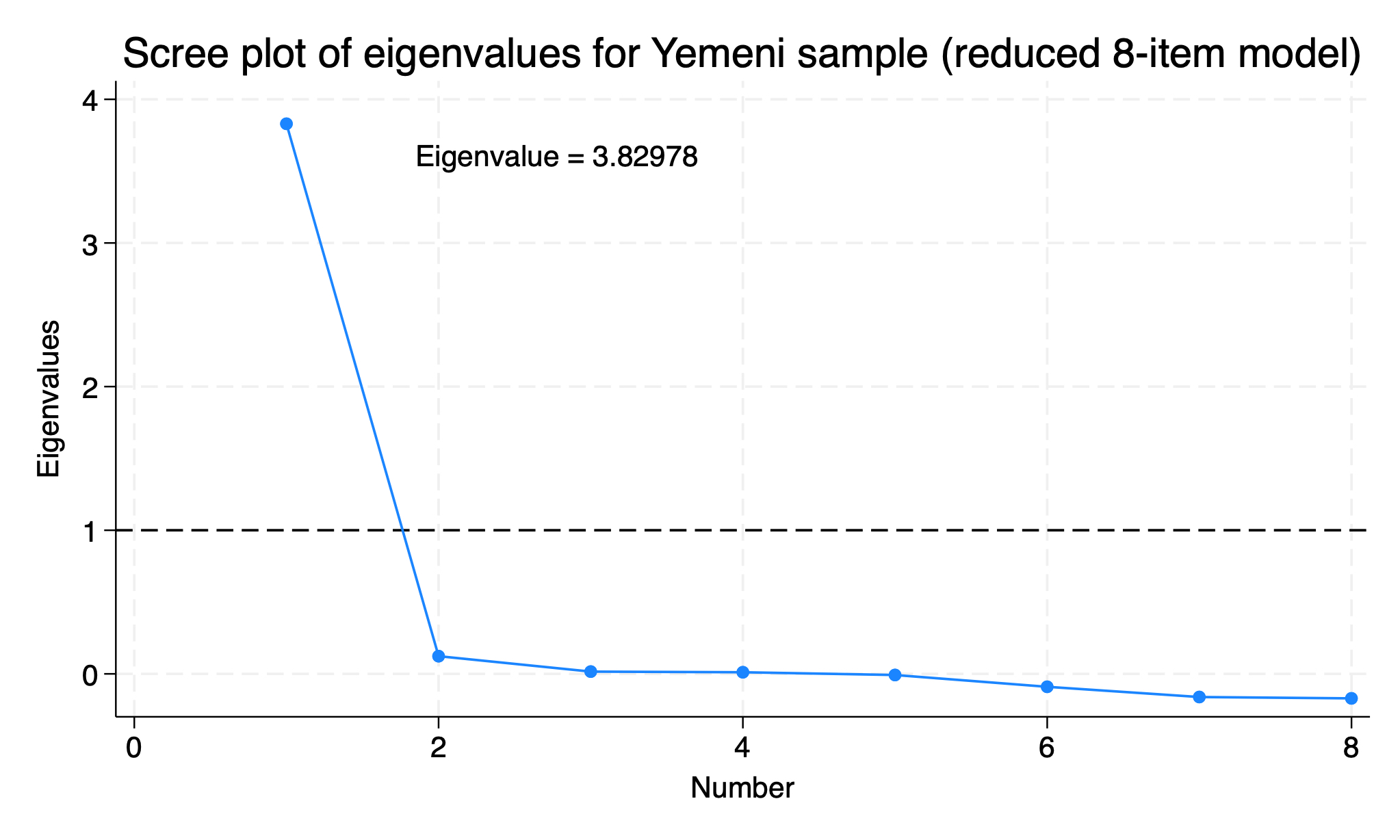

Supplement: S1 Fig — (TIFF) [file pone.0352966.s004.tiff]

Factor Loadings of Attitude Items (Yemeni Sample)

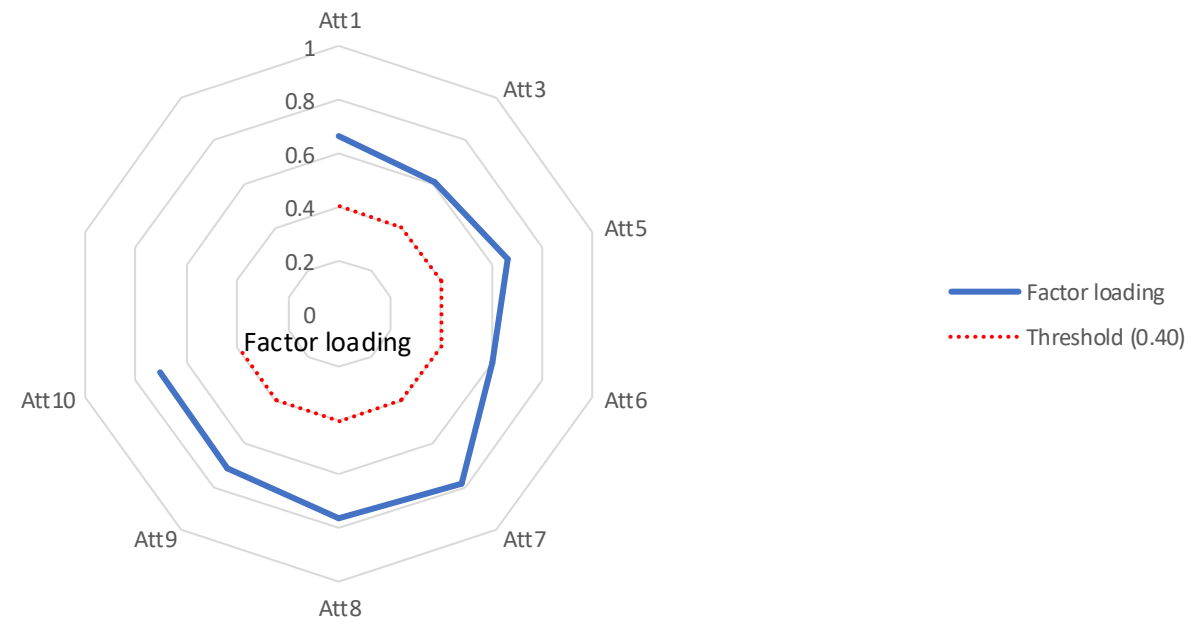

Supplement: S2 Fig — The red line represents the loading threshold of 0.40. All items exceeded this threshold, indicating adequate contribution to the underlying construct. (PDF) [file pone.0352966.s005.pdf]
