# Supplementary material for: Cross-cultural assessment of knowledge and attitudes toward Folic acid: Instrument development and validation in Thailand and Yemen
Source: PLoS One. 2026 Jul 15;21(7):e0352966. doi: 10.1371/journal.pone.0352966 (PMC13372155; doi:10.1371/journal.pone.0352966)
Supplement: S1 Questionnaire — (DOCX) [file pone.0352966.s006.docx]

**แบบสอบถามเรื่องความรู้และทัศนคติต่อการบริโภคกรดโฟลิก**

**ตอนที่ 1: ข้อมูลทั่วไป**

1. เพศ

□ หญิง

2. อายุ

……………… ปี

3. สถานภาพสมรส

□ โสด

□ สมรส – ไม่มีบุตร

□ สมรส – มีบุตร

□ หย่าร้าง – ไม่มีบุตร

□ หย่าร้าง – มีบุตร

□ อื่น ๆ ……………………………

4. ระดับการศึกษาในปัจจุบัน

(เลือกเพียงข้อเดียว)

□ ต่ำกว่ามัธยมศึกษาตอนปลาย

□ ประกาศนียบัตรวิชาชีพ หรือเทียบเท่า

□ ปริญญาตรี

□ ศึกษาต่อด้านการแพทย์

□ ไม่ประสงค์ตอบ

5. อาชีพ

(เลือกเพียงข้อเดียว)

□ นักศึกษา

□ ข้าราชการ/พนักงานรัฐ

□ พนักงานบริษัทเอกชน

□ ทำงานพาร์ทไทม์

□ แม่บ้าน

□ ว่างงาน

□ ไม่ประสงค์ตอบ

6. ท่านเคยได้ยินเกี่ยวกับ “กรดโฟลิก (Folic acid)” มาก่อนหรือไม่?

□ เคย

□ ไม่เคย

**ตอนที่ 2: ข้อคำถามด้านความรู้ (Knowledge questions)**

*(ตอบเพียงหนึ่งข้อในแต่ละคำถาม)*

1. ประเทศไทยมีเด็กพิการแต่กำเนิดประมาณ 30,000 คนต่อปี

□ ถูก

□ ผิด

□ ไม่แน่ใจ/ไม่ทราบ

1. ภาวะปากแหว่งเพดานโหว่ไม่ถือเป็นความพิการแต่กำเนิด

□ ถูก

□ ผิด

□ ไม่แน่ใจ/ไม่ทราบ

1. Folic acid พบในอาหารธรรมชาติ เช่น ผักใบเขียว ไข่แดง ตับ ถั่ว รวมถึงอาหารเสริมและวิตามิน

□ ถูก

□ ผิด

□ ไม่แน่ใจ/ไม่ทราบ

1. ปัจจุบันหลายประเทศผสม Folic acid ลงในอาหาร เช่น ข้าว

□ ถูก

□ ผิด

□ ไม่แน่ใจ/ไม่ทราบ

1. ควรบริโภค Folic acid ตั้งแต่ก่อนตั้งครรภ์จนถึง 3 เดือนแรกของการตั้งครรภ์

□ ถูก

□ ผิด

□ ไม่แน่ใจ/ไม่ทราบ

1. ผู้หญิงตั้งครรภ์ควรบริโภค Folic acid 5 มิลลิกรัมต่อวัน เพื่อลดความเสี่ยงทารกพิการแต่กำเนิด

□ ถูก

□ ผิด

□ ไม่แน่ใจ/ไม่ทราบ

1. Folic acid ไม่สามารถขับออกจากร่างกายได้

□ ถูก

□ ผิด

□ ไม่แน่ใจ/ไม่ทราบ

1. ผู้หญิงวัยเจริญพันธุ์เท่านั้นที่สามารถรับประทาน Folic acid ได้

□ ถูก

□ ผิด

□ ไม่แน่ใจ/ไม่ทราบ

**ตอนที่ 3: ข้อคำถามด้านทัศนคติ (Attitude questions)**

*ใช้มาตราส่วน 5 ระดับ (1 ไม่เห็นด้วยอย่างยิ่ง – 5 เห็นด้วยอย่างยิ่ง)*

15. ท่านเห็นสมควรว่าผู้หญิงวัยเจริญพันธุ์ควรบริโภค Folic acid

1 □ 2 □ 3 □ 4 □ 5 □

16. ท่านจะเลือกรับประทานอาหาร/เครื่องดื่มที่ผสม Folic acid แม้มีราคาแพงกว่า

1 □ 2 □ 3 □ 4 □ 5 □

17. หากมีคนรู้จักแนะนำให้บริโภค Folic acid ท่านจะไม่ลังเลที่จะปฏิบัติตาม

1 □ 2 □ 3 □ 4 □ 5 □

18. ท่านเห็นด้วยหากมีกฎหมายบังคับให้ผสม Folic acid ลงในข้าว

1 □ 2 □ 3 □ 4 □ 5 □

19. ท่านคิดว่าการบริโภค Folic acid ตั้งแต่ก่อนตั้งครรภ์ถึง 3 เดือนแรก เป็นผลดีมากกว่าผลเสีย

1 □ 2 □ 3 □ 4 □ 5 □

20. ท่านคิดว่าการบริโภค Folic acid ระหว่างตั้งครรภ์สามารถป้องกันความพิการแต่กำเนิดได้

1 □ 2 □ 3 □ 4 □ 5 □

21. หากมีแผนตั้งครรภ์ ท่านจะซื้อ Folic acid มารับประทาน

1 □ 2 □ 3 □ 4 □ 5 □

22. ท่านเห็นด้วยหากมีการแจก Folic acid ฟรีแก่หญิงวัยเจริญพันธุ์

1 □ 2 □ 3 □ 4 □ 5 □

23. ท่านคิดว่า Folic acid สามารถหาซื้อได้ตามร้านขายยาทั่วไป

1 □ 2 □ 3 □ 4 □ 5 □

24. ท่านเห็นด้วยหากรัฐบาลสนับสนุนให้ประชาชนเข้าถึง Folic acid ได้ง่ายขึ้น

1 □ 2 □ 3 □ 4 □ 5 □
