# Supplementary material for: Cross-cultural assessment of knowledge and attitudes toward Folic acid: Instrument development and validation in Thailand and Yemen
Source: PLoS One. 2026 Jul 15;21(7):e0352966. doi: 10.1371/journal.pone.0352966 (PMC13372155; doi:10.1371/journal.pone.0352966)
Supplement: S2 Questionnaire — (DOCX) [file pone.0352966.s007.docx]

**استبيان حول المعرفة والمواقف تجاه استهلاك حمض الفوليك**

## الجزء 1: المعلومات العامة

1. **الجنس**

□ أنثى

1. **العمر**

……………… سنة

1. **الحالة الاجتماعية**

□ عازبة

□ متزوجة – ليس هنالك أطفال

□ متزوجة – لدي أطفال

□ مطلقة – ليس هنالك أطفال

□ مطلقة – لدي أطفال

□ أخرى: ……………………………

1. **المستوى التعليمي الحالي**

حدد دائرة واحدة فقط

□ ثالث ثانوي أو ما يعادل هذا المستوى

□ دبلوم أو حاصلة على درجة علمية عالية

□ حاصلة على درجة بكالوريوس

□ دراسات عليا

□ أفضل أن لا أجيب

1. **المهنة**

حدد دائرة واحدة فقط

□ طالبة

□ موظفة حكومية

□ موظفة غير حكومية

□ دوام جزئي

□ ربة أسرة

□ عاطلة عن العمل

□ أفضل أن لا أجيب

1. **هل سمعتِ عن حمض الفوليك (فيتامين B9) من قبل؟**

□ نعم

□ لا

## الجزء 2: أسئلة المعرفة (8 أسئلة)

### (الرجاء اختيار خيار واحد فقط لكل سؤال)

1. يولد حوالي 240 ألف طفل مصابين بتشوهات خلقية كل عام

□ صحيح

□ خطأ

□ لست متأكدة / لا أعلم

1. لا تُعتبر الشفة الأرنبية وشق سقف الحلق عيباً خلقياً.

□ صحيح

□ خطأ

□ لست متأكدة / لا أعلم

1. يتواجد حمض الفوليك في الأطعمة الطبيعية مثل الخضار الورقية، صفار البيض، الكبد، الفاصولياء، وكذلك في المكملات الغذائية والفيتامينات.

□ صحيح

□ خطأ

□ لست متأكدة / لا أعلم

1. حالياً، هناك العديد من الدول التي تضيف حمض الفوليك إلى المواد الغذائية الأساسية مثل الأرز

□ صحيح

□ خطأ

□ لست متأكدة / لا أعلم

1. يجب تناول حمض الفوليك قبل فترة الحمل وحتى الأشهر الثلاثة الأولى من الحمل.

□ صحيح

□ خطأ

□ لست متأكدة / لا أعلم

1. للحد من مخاطر العيوب الخلقية، ينبغي على النساء الحوامل تناول 5 ملغ من حمض الفوليك يومياً.

□ صحيح

□ خطأ

□ لست متأكدة / لا أعلم

1. لا يمكن إخراج حمض الفوليك من الجسم.

□ صحيح

□ خطأ

□ لست متأكدة / لا أعلم

1. يمكن للنساء تناول حمض الفوليك في سن الإنجاب فقط.

□ صحيح

□ خطأ

□ لست متأكدة / لا أعلم

الجزء 3: التوجهات تجاه استهلاك حمض الفوليك (10 أسئلة)

يرجى اختيار إجابة واحدة لكل سؤال حسب مقياس ليكرت من خمس درجات:

١ – موافق بشدة

٢ – موافق

٣ – لست متأكد/ة

٤ – لا أوافق

٥ – لا أوافق بشدة

(الرجاء اختيار خيار واحد فقط لكل سؤال)

.15النساء اللواتي في سن الإنجاب يجب عليهن تناول حمض الفوليك.

٥ ☐ ٤ ☐ ٣ ☐ ٢ ☐ ١ ☐

1. ستختارين الأطعمة التي تحتوي على حمض الفوليك، رغم أنها أغلى ثمناً.

٥ ☐ ٤ ☐ ٣ ☐ ٢ ☐ ١ ☐

1. إذا نُصِحتِ باستخدام حمض الفوليك، فلن تترددي في اتباع النصيحة.

٥ ☐ ٤ ☐ ٣ ☐ ٢ ☐ ١ ☐

1. يجب أن يكون هناك قانون يفرض إضافة حمض الفوليك إلى المواد الغذائية الأساسية مثل الخبز والأرز.

٥ ☐ ٤ ☐ ٣ ☐ ٢ ☐ ١ ☐

1. تناول حمض الفوليك قبل الحمل وحتى الأشهر الثلاثة الأولى نافع أكثر من كونه ضاراً.

٥ ☐ ٤ ☐ ٣ ☐ ٢ ☐ ١ ☐

1. استهلاك حمض الفوليك أثناء الحمل قد يمنع التشوهات الخلقية.

٥ ☐ ٤ ☐ ٣ ☐ ٢ ☐ ١ ☐

1. إذا كنتِ تخططين للحمل، فسوف تشترين وتتناولين حمض الفوليك.

٥ ☐ ٤ ☐ ٣ ☐ ٢ ☐ ١ ☐

1. تقديم حمض الفوليك مجاناً للنساء في سن الإنجاب فكرة مناسبة.

٥ ☐ ٤ ☐ ٣ ☐ ٢ ☐ ١ ☐

1. حمض الفوليك متوفر بسهولة في معظم الصيدليات.

٥ ☐ ٤ ☐ ٣ ☐ ٢ ☐ ١ ☐

1. توافقين على برنامج الحكومة لدعم توزيع حمض الفوليك لتحسين وصوله إلى المجتمع.

٥ ☐ ٤ ☐ ٣ ☐ ٢ ☐ ١ ☐
