# Supplementary material for: Cross-cultural assessment of knowledge and attitudes toward Folic acid: Instrument development and validation in Thailand and Yemen
Source: PLoS One. 2026 Jul 15;21(7):e0352966. doi: 10.1371/journal.pone.0352966 (PMC13372155; doi:10.1371/journal.pone.0352966)
Supplement: S3 Questionnaire — (DOCX) [file pone.0352966.s008.docx]

**Questionnaire on Knowledge and Attitudes Toward Folic Acid Consumption**

**Part 1: Demographic Information**

1. Sex

☐ Female

2. Age

……………… years

3. Marital status

□ Single

□ Married – no children

□ Married – with children

□ Divorced – no children

□ Divorced – with children

□ Other: ……………………………

4. Current level of education

(Select one)

□ Below high school

□ Vocational certificate or equivalent

□ Bachelor’s degree

□ Medical-related studies

□ Prefer not to answer

5. Occupation

(Select one)

□ Student

□ Government employee / public sector

□ Private sector employee

□ Part-time worker

□ Homemaker

□ Unemployed

□ Prefer not to answer

6. Have you ever heard of folic acid?

□ Yes

□ No

**Part 2: Knowledge questions**

(Select one answer for each question)

7. Approximately 30,000 children are born with congenital disabilities each year in Thailand.

□ True

□ False

□ Not sure / Do not know

8. Cleft lip and cleft palate are not considered congenital disabilities.

□ True

□ False

□ Not sure / Do not know

1. Folic acid is found in natural foods such as leafy green vegetables, egg yolks, liver, and beans, as well as in dietary supplements and vitamins.

□ True

□ False

□ Not sure / Do not know

1. Currently, many countries add Folic acid to staple foods such as rice.

□ True

□ False

□ Not sure / Do not know

1. Folic acid should be consumed from the pre-pregnancy period through the first three months of pregnancy.

□ True

□ False

□ Not sure / Do not know

1. Pregnant women should take 5 mg of folic acid per day to reduce the risk of congenital disabilities in infants.

□ True

□ False

□ Not sure / Do not know

13. Folic acid cannot be excreted from the body.

□ True

□ False

□ Not sure / Do not know

14. Only women of reproductive age can take folic acid.

□ True

□ False

□ Not sure / Do not know

**Part 3: Attitude questions**

Using a 5-point Likert scale (1 = Strongly disagree – 5 = Strongly agree)

15. Women of reproductive age should take folic acid.

1 □ 2 □ 3 □ 4 □ 5 □

16. I would choose foods or beverages fortified with folic acid, even if they are more expensive.

1 □ 2 □ 3 □ 4 □ 5 □

17. If someone I know recommends taking folic acid, I would follow the advice without hesitation.

1 □ 2 □ 3 □ 4 □ 5 □

18. I would support a law requiring the addition of folic acid to staple foods such as rice.

1 □ 2 □ 3 □ 4 □ 5 □

19. Taking folic acid from before pregnancy until the first three months of pregnancy has more benefits than risks.

1 □ 2 □ 3 □ 4 □ 5 □

20. Taking folic acid during pregnancy can prevent congenital disabilities.

1 □ 2 □ 3 □ 4 □ 5 □

21. If I were planning a pregnancy, I would buy and take folic acid.

1 □ 2 □ 3 □ 4 □ 5 □

22. I would support providing free folic acid to women of reproductive age.

1 □ 2 □ 3 □ 4 □ 5 □

23. I believe that folic acid is readily available at pharmacies.

1 □ 2 □ 3 □ 4 □ 5 □

24. I would support government efforts to improve access to folic acid.

1 □ 2 □ 3 □ 4 □ 5 □
